# First-line durvalumab plus platinum-etoposide in extensive-stage small-cell lung cancer: CASPIAN Japan subgroup analysis

**DOI:** 10.1007/s10147-021-01899-8

**Published:** 2021-04-07

**Authors:** Katsuyuki Hotta, Makoto Nishio, Haruhiro Saito, Isamu Okamoto, Yasuharu Nakahara, Hidetoshi Hayashi, Manabu Hayama, Peter Laud, Haiyi Jiang, Luis Paz-Ares, Koichi Azuma

**Affiliations:** 1 Department of Respiratory Medicine, Okayama University Hospital, 2-5-1 Shikata-cho, Kita-ku, Okayama, 700 8558 Japan; 2 Center for Innovative Clinical Medicine, Okayama University Hospital, 2-5-1 Shikata-cho, Kita-ku, Okayama, 700 8558 Japan; 3 Department of Thoracic Medical Oncology, The Cancer Institute Hospital, Japanese Foundation for Cancer Research, Tokyo, Japan; 4 Department of Thoracic Oncology, Kanagawa Cancer Center, Yokohama, Japan; 5 Research Institute for Diseases of the Chest, Graduate School of Medical Sciences, Kyushu University, Fukuoka, Japan; 6 Department of Respiratory Medicine, Himeji Medical Center, Himeji, Japan; 7 Department of Medical Oncology, Kindai University Faculty of Medicine, Osaka-Sayama, Japan; 8 AstraZeneca K.K, Tokyo, Japan; 9 Statistical Services Unit, University of Sheffield, Sheffield, UK; 10 AstraZeneca, Gaithersburg, MD USA; 11 Department of Medical Oncology, Hospital Universitario 12 de Octubre, Madrid, Spain; 12 Department of Internal Medicine, Kurume University School of Medicine, Kurume, Japan

**Keywords:** Small-cell lung cancer, Durvalumab, CASPIAN, Japanese patients, Subgroup analysis

## Abstract

**Background:**

In the phase 3 CASPIAN study (NCT03043872), first-line durvalumab plus etoposide and cisplatin or carboplatin (EP) significantly improved OS versus EP alone in patients with extensive-stage (ES)-SCLC (HR 0.73 [95% CI 0.59–0.91; *p* = 0.0047]). Here we report results for a preplanned subgroup analysis of patients recruited in Japan.

**Methods:**

Treatment-naïve patients with ES-SCLC received either 4 cycles of durvalumab 1500 mg plus EP q3w followed by maintenance durvalumab 1500 mg q4w until disease progression or up to 6 cycles of EP q3w. The primary endpoint was OS. Secondary endpoints included progression-free survival (PFS), objective response rate (ORR), safety, and tolerability.

**Results:**

In the Japan subgroup, 18 patients were randomized to durvalumab plus EP and 16 patients to EP. At the interim analysis with a median follow-up of 12.5 months in the subgroup, OS numerically favored durvalumab plus EP versus EP (HR 0.77 [95% CI 0.26‒2.26]; median not reached vs 15.2 months). PFS was similar for durvalumab plus EP versus EP (HR 0.90 [95% CI 0.43‒1.89]). Confirmed ORR was 89% with durvalumab plus EP versus 69% with EP. Adverse events (AEs) of CTCAE grade 3 or 4 were reported in 78% versus 94% of patients in the durvalumab plus EP versus EP arms. There were no AEs leading to treatment discontinuation or death in the Japan subgroup.

**Conclusion:**

First-line durvalumab plus EP was effective and well tolerated in Japanese patients with ES-SCLC. Despite the small size of the Japan subgroup, results were generally consistent with the global study population.

**Supplementary Information:**

The online version contains supplementary material available at 10.1007/s10147-021-01899-8.

## Introduction

Small-cell lung cancer (SCLC) accounts for approximately 10% of all lung cancers in Japan, with a decline in incidence since the 1990s [1, 2]. Approximately 40‒50% of Japanese patients diagnosed with SCLC have extensive-stage SCLC (ES-SCLC), of whom less than 4% are alive 5 years after diagnosis [1, 3]. Over the last three decades, first-line standard of care (SoC) treatment for ES-SCLC in Japan has primarily consisted of etoposide in combination with either carboplatin or cisplatin (EP) [4]; cisplatin plus irinotecan is also recommended by the Japanese Lung Cancer Society Guidelines for patients aged 70 years or younger with performance status 0–2 and without comorbidities contraindicating use of irinotecan [5]. Two global, phase 3 studies have recently shown that the addition of immunotherapy targeting the programmed cell death-1 (PD-1)/programmed cell death ligand-1 (PD-L1) pathway to platinum-based chemotherapy (atezolizumab in combination with etoposide plus carboplatin [6] or durvalumab in combination with etoposide plus investigator’s choice of either carboplatin or cisplatin [7]) improved overall survival (OS) in the first-line setting.

Durvalumab is a selective, high-affinity human IgG1 monoclonal antibody that blocks binding of PD-L1 to PD-1 and CD80 [8]. The ongoing phase 3 CASPIAN study (NCT03043872) is investigating the efficacy and safety of first-line durvalumab, with or without the anti-cytotoxic T lymphocyte-associated antigen-4 (CTLA-4) antibody tremelimumab, in combination with EP, compared with EP alone in patients with ES-SCLC [7]. Durvalumab plus EP significantly improved OS versus EP at the planned interim analysis (data cut-off: March 11, 2019; 63% maturity), with a hazard ratio (HR) of 0.73 (95% CI 0.59‒0.91; *p* = 0.0047; median OS, 13.0 months vs 10.3 months in the durvalumab plus EP and EP arms, respectively) [7]. This is therefore considered as the final result, in terms of formal statistical testing, for durvalumab plus EP versus EP. The survival benefit was seen across all prespecified patient subgroups and progression-free survival (PFS) also favored durvalumab plus EP, with a HR of 0.78 (95% CI 0.65‒0.94); in the durvalumab plus EP versus EP arms, median PFS was 5.1 months versus 5.4 months and 12-month PFS was 18% versus 5%. Safety findings were consistent with the known safety profiles of durvalumab and EP. The durvalumab plus tremelimumab plus EP arm had not met the predefined statistical significance threshold at the time of the planned interim analysis and therefore the sponsor remained blinded to this arm, which continued to the final analysis. Based on the positive results from CASPIAN, durvalumab was recently approved in several countries, including the USA, Japan, the EU, and other countries globally as a first-line treatment for ES-SCLC in combination with EP [9].

As the use of immune checkpoint inhibitors such as durvalumab increases across the globe, it is important to assess the activity of each agent across different ethnic patient groups. Differences in OS and toxicity with anticancer therapy have been previously reported between Asian and Caucasian patients with both SCLC and non-small cell lung cancer (NSCLC) [10–12], although to date the pharmacokinetics, safety, and efficacy of immune checkpoint inhibitors, including durvalumab, appear similar in Japanese and non-Japanese patients with solid tumors including SCLC [13–18].

We report a preplanned subgroup analysis (data cut-off: March 11, 2019) from the CASPIAN study assessing the efficacy and safety of durvalumab plus EP compared with EP alone as first-line treatment for patients with ES-SCLC recruited in Japan.

## Patients and methods

### Study design and patients

CASPIAN is an open-label, sponsor-blind, multicenter, randomized, phase 3 study taking place at centers across Europe, North and South America, and Asia, including 19 sites in Japan. Efficacy and safety results from the global study population at the planned interim analysis have been previously reported, along with detailed study methodology including full eligibility criteria [7]. In brief, the study population comprised patients with treatment-naïve, histologically or cytologically documented ES-SCLC, who were aged ≥ 18 years (≥ 20 years for Japanese patients), had a World Health Organization (WHO) performance status score of 0 or 1, bodyweight of at least 30 kg, and measurable disease according to Response Evaluation Criteria in Solid Tumors (RECIST), version 1.1 [19]. Patients with brain metastases were eligible provided that they were either asymptomatic or treated and stable, and had been off steroids and anticonvulsants for at least 1 month before study entry. Patients were excluded if they had a history of radiotherapy to the chest, or planned consolidation chest radiotherapy; active or previous autoimmune or inflammatory disorders; paraneoplastic syndrome of autoimmune nature requiring systemic treatment; a history of active primary immunodeficiency; or uncontrolled, concurrent illness or active infections.

All patients provided signed informed consent for participation in the study. The study protocol and all modifications were approved by the relevant ethics committees and regulatory authorities, and the study was run in accordance with the International Conference on Harmonisation good clinical practice guidelines, the Declaration of Helsinki, and applicable local regulations. Periodic safety monitoring and the planned interim efficacy assessment were conducted by an independent data monitoring committee.

### Treatment

Patients were randomized in a 1:1:1 ratio to receive durvalumab plus EP, durvalumab plus tremelimumab plus EP, or EP; randomization was stratified by planned platinum agent (carboplatin or cisplatin). EP in all arms comprised etoposide 80‒100 mg/m^2^, administered on days 1‒3 of each 21-day cycle, and investigator’s choice of either carboplatin area under the curve 5‒6 mg/mL/min or cisplatin 75‒80 mg/m^2^, administered on day 1 of each cycle. Patients in the immunotherapy arms received durvalumab 1500 mg, with or without tremelimumab 75 mg, on day 1 of each cycle, plus EP every 3 weeks for 4 cycles, followed by maintenance durvalumab 1500 mg every 4 weeks. Patients in the EP arm could receive up to 6 cycles of EP, as well as prophylactic cranial irradiation administered post-EP at the investigator’s discretion.

Treatment continued until disease progression per investigator assessment, unacceptable toxicity, or other discontinuation criteria were met. Study treatment could be continued beyond disease progression if the investigator judged a patient to be deriving clinical benefit.

### Endpoints and assessments

The primary endpoint was OS. Secondary endpoints included PFS and unconfirmed objective response rate based on investigator assessment according to RECIST v1.1, and safety and tolerability. In addition, symptoms and health-related quality of life assessments were a prespecified secondary endpoint and have been reported in the global population [20]. Confirmed objective response rate and duration of confirmed response were analyzed post hoc. Evaluation of efficacy and safety in patients recruited in Japan (hereafter referred to as the Japan subgroup) was a preplanned analysis, to assess the benefit‒risk for this population and consistency with the global population.

Tumor imaging was performed every 6 weeks for the first 12 weeks, and then every 8 weeks, until confirmed objective disease progression. Survival was monitored every 2 months after treatment discontinuation. Adverse events were graded per National Cancer Institute Common Terminology Criteria for Adverse Events, version 4.03.

### Statistical analysis

Full details of the statistical analysis have been reported previously [7]. In brief, approximately 795 patients were to be randomized, with the final OS analysis planned at 80% maturity. The interim OS analysis was planned after approximately 318 events had occurred both in the combined durvalumab plus EP and EP arms and in the combined durvalumab plus tremelimumab plus EP and EP arms (60% maturity).

OS and PFS in the Japan subgroup were analyzed using an unstratified log-rank test, with HRs and 95% CIs estimated using an unstratified Cox proportional hazards model and ties handled by the Efron approach. The Kaplan–Meier method was used to calculate medians, and 95% CIs for the medians were derived based on the Brookmeyer–Crowley method and using the log–log transformation.

The objective response rate was compared between treatment arms using an unstratified logistic regression model, with 95% CIs calculated by profile likelihood. Duration of response was calculated using the Kaplan–Meier method.

Data underlying the findings described in this manuscript may be obtained in accordance with AstraZeneca’s data sharing policy described at: https://astrazenecagrouptrials.pharmacm.com/ST/Submission/Disclosure

## Results

### Patients and treatment

Patients were enrolled in the CASPIAN study between March 2017 and May 2018. A total of 34 patients were randomized to the durvalumab plus EP and EP arms in the Japan subgroup (18 and 16 patients, respectively). All 34 patients were treated and were included in both efficacy and safety analyses of the Japan subgroup. The overall median age of the Japan subgroup was 69.0 years (range 40‒82); most patients were men (82%) and former smokers (91%) (Table 1). There were numerical differences in baseline demographics and disease characteristics between treatment arms, as might be expected given the small number of patients in each treatment arm (Table 1).

**Table 1:** Baseline patient demographics and disease characteristics

	Durvalumab + EP (*n* = 18)	EP (*n* = 16)	All patients (*n* = 34)
Median age (range), years	67.5 (40–82)	69.5 (46–82)	69 (40–82)
Age group, *n* (%)			
< 65	5 (28)	5 (31)	10 (29)
≥ 65	13 (72)	11 (69)	24 (71)
Sex, *n* (%)			
Men	14 (78)	14 (88)	28 (82)
Women	4 (22)	2 (13)	6 (18)
Median body weight (range), kg	58.0 (50.0–93.0)	60.0 (41.0–91.0)	58.5 (41.0–93.0)
Disease stage, *n* (%)			
IIIB	3 (17)	1 (6)	4 (12)
IV	15 (83)	15 (94)	30 (88)
WHO performance status, *n* (%)			
0	7 (39)	4 (25)	11 (32)
1	11 (61)	12 (75)	23 (68)
Smoking history, *n* (%)			
Never smoker	0	0	0
Former smoker	17 (94)	14 (88)	31 (91)
Current smoker	1 (6)	2 (13)	3 (9)
Brain or CNS metastases, *n* (%)	1 (6)	3 (19)	4 (12)
Liver metastases, *n* (%)	10 (56)	7 (44)	17 (50)

*CNS* central nervous system, *EP* platinum-etoposide, *WHO* World Health Organization

In the durvalumab plus EP and EP arms, 11 (61%) and 8 (50%) patients, respectively, received carboplatin and 7 (39%) and 8 (50%) patients received cisplatin (Table 2). Patients in the durvalumab plus EP arm received a median of 6 (range 3‒14) doses of durvalumab and a median of 4 (range 3‒4) cycles of EP (Table 2). Seventeen (94%) patients received the maximum permitted 4 cycles of EP; one patient discontinued EP early because of disease progression. Patients in the EP arm received a median of 4 (range 2‒6) cycles of EP (Table 2). Thirteen (81%) patients received at least 4 cycles and 6 (38%) patients received 6 cycles of EP. Twelve (75%) patients completed their planned number of cycles of EP; 4 (25%) patients discontinued EP early because they withdrew their consent (*n* = 2), had progressive disease (*n* = 1), or developed other withdrawal criteria (*n* = 1; due to risk of ileus relapse and general worsening of condition). At the time of data cut-off, one patient (6%) remained on durvalumab treatment and 17 (94%) patients had discontinued durvalumab, all attributable to disease progression; none of the patients in the EP arm remained on treatment.

**Table 2:** Treatment exposure

	Durvalumab + EP (*n* = 18)	EP (*n* = 16)
Median number (range) of durvalumab doses	6 (3–14)	–
Median (range) total duration of durvalumab, weeks	20.6 (10.3–75.0)	–
Platinum agent received, *n* (%)		
Carboplatin	11 (61)	8 (50)
Cisplatin	7 (39)	8 (50)
Median number (range) of cycles of EP^a^	4 (3–4)	4 (2–6)
Cycles of EP received, *n* (%)^a^		
≥ 4	17 (94)	13 (81)
≥ 5	0	6 (38)
6	0	6 (38)
Median (range) total duration of EP, weeks^a^	12.6 (10.0–14.7)	13.1 (6.7–24.7)

*EP* platinum-etoposide

^a^Based on etoposide exposure

In the durvalumab plus EP and EP arms, respectively, 13/18 (72%) patients and 13/16 (81%) patients received one or more subsequent systemic anticancer therapies, which in the majority of cases was chemotherapy. Eight (44%) and 5 (31%) patients, respectively, received ≥ 2 lines of subsequent systemic anticancer therapy (Supplementary Table 1). No patients in the Japan subgroup received prophylactic cranial irradiation consistent with treatment practice patterns in Japan [21].

### Efficacy

**Fig. 1:**
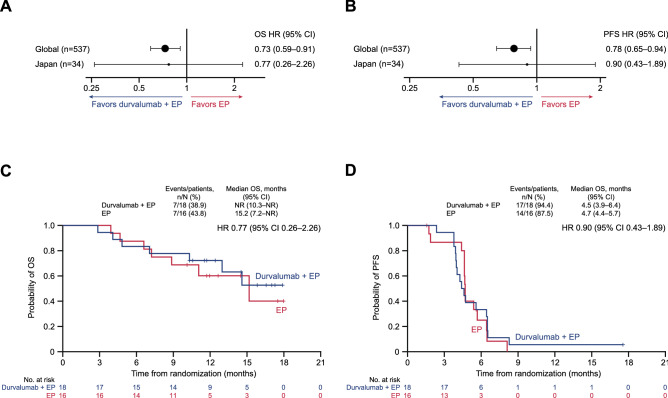
OS and investigator-assessed PFS. Forest plots of OS (**a**) and PFS (**b**) HRs for the Japan subgroup and global population and Kaplan–Meier graphs of OS (**c**) and PFS (**d**) in the Japan subgroup. *EP* platinum-etoposide, *HR* hazard ratio, *NR* not reached, *OS* overall survival, *PFS* progression-free survival

At the time of data cut-off, the median duration of follow-up for OS in censored patients in the Japan subgroup was 12.5 months (range 10.1‒18.0). Seven patients had died in each treatment arm (41% maturity) and the remaining 20 patients were still being followed for survival. OS numerically favored durvalumab plus EP versus EP, with an HR of 0.77 (95% CI 0.26‒2.26); median OS was not reached (95% CI 10.3 months‒not reached) in the durvalumab plus EP arm and was 15.2 months (95% CI 7.2‒not reached) in the EP arm (Fig. 1a and c). The estimated OS rate at 12 months was 72.2% (95% CI 45.6‒87.4) in the durvalumab plus EP arm compared with 60.2% (95% CI 31.2‒80.1) in the EP arm.

Seventeen (94%) patients in the durvalumab plus EP arm and 14 (88%) patients in the EP arm had experienced disease progression or died by the time of data cut-off. PFS was similar for durvalumab plus EP versus EP (HR, 0.90 [95% CI 0.43‒1.89]), with median PFS of 4.5 months (95% CI 3.9‒6.4) versus 4.7 months (95% CI 4.4‒5.7) (Fig. 1b and d). The PFS rate at 6 months was 33.3% (95% CI 13.7‒54.5) with durvalumab plus EP versus 25.0% (95% CI 6.9‒48.8) with EP.

**Fig. 2:**
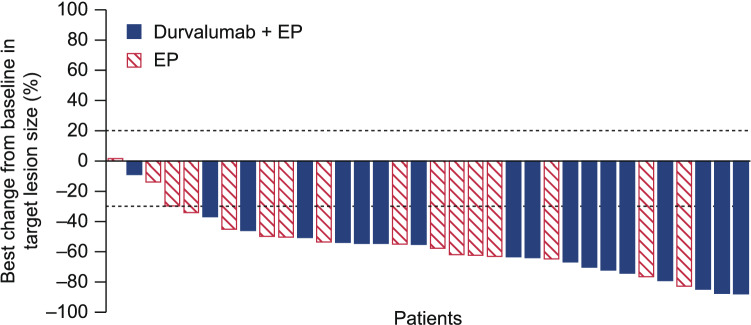
Best percentage change from baseline in target lesion size for patients receiving treatment with durvalumab + EP or EP. Target lesion size based on site investigator assessment according to RECIST v1.1 Dashed reference lines at − 30% and + 20% indicate thresholds for partial response and disease progression, respectively

Investigator-assessed confirmed objective responses were achieved in 16 (89%) of 18 patients in the durvalumab plus EP arm compared with 11 (69%) of 16 patients in the EP arm (odds ratio 3.64 [95% CI 0.65‒28.75]) (Table 3). There were no complete responses in the Japan subgroup. The median (range) best reduction from baseline in target lesion size was − 63.55% (− 87.7 to − 8.9) in the durvalumab plus EP arm compared with − 53.90% (− 82.4 to 1.6) in the EP arm. The depth of response is shown in Fig. 2. Among patients with a confirmed response, the median duration of response was similar in each treatment arm (Table 3).

**Table 3:** Summary of tumor response

	Durvalumab + EP (*n* = 18)	EP (*n* = 16)
Unconfirmed objective response, *n* (%)^a^	17 (94)	13 (81)
Odds ratio (95% CI)	3.92 (0.44–84.49)	
Confirmed objective response, *n* (%)^a^	16 (89)	11 (69)
Odds ratio (95% CI)	3.64 (0.65–28.75)	
Best unconfirmed response, *n* (%)^a^		
Complete	0	0
Partial	17 (94)	13 (81)
Stable disease ≥ 6 weeks	1 (6)	3 (19)
Progressive disease	0	0
Best reduction from baseline in target lesion size, %^a^		
Mean ± SD	– 61.6 ± 19.59	– 49.6 ± 21.88
Median (range)	– 63.55 (– 87.7 to – 8.9)	– 53.90 (– 82.4 to 1.6)
Median (95% CI) duration of response, months^b^	3.1 (2.5–5.1)	3.5 (3.3–5.0)
Remaining in response, %^b^		
At 6 months	12.5	9.1
At 12 months	6.3	0

*EP* platinum-etoposide, *RECIST* Response Evaluation Criteria in Solid Tumors

^a^Investigator-assessed objective response per RECIST v1.1

^b^Calculated using the Kaplan–Meier method; based on confirmed responses

### Safety

Adverse events (AEs) were reported in all patients, while grade 3 or 4 AEs were reported in 14 (78%) of 18 patients treated with durvalumab plus EP and 15 (94%) of 16 patients treated with EP (Table 4). Across both treatment arms, the most common grade 3 or 4 AEs (neutropenia, febrile neutropenia, neutrophil count decreased, and white blood cell count decreased) were generally associated with chemotherapy (Table 5). Serious AEs occurred in 8 (44%) patients in the durvalumab plus EP arm and 8 (50%) patients in the EP arm (Table 4) and were most commonly hematological toxicities and infections. The most common serious AE was febrile neutropenia, occurring in 2 patients in each arm (11% in the durvalumab plus EP arm and 13% in the EP arm; Supplementary Table 2).

**Table 4:** Summary of safety in the Japan subgroup and the global study population

	Japan subgroup		Global study population	
	Durvalumab + EP (*n* = 18)	EP (*n* = 16)	Durvalumab + EP (*n* = 265)	EP (*n* = 266)
Any event of any cause, *n* (%)	18 (100)	16 (100)	260 (98)	258 (97)
Any grade 3 or 4 event	14 (78)	15 (94)	163 (62)	166 (62)
Any event leading to death	0	0	13 (5)	15 (6)
Any serious event	8 (44)	8 (50)	82 (31)	96 (36)
Any event leading to discontinuation^a^	0	0	25 (9)	25 (9)
Any immune-mediated event^b^	4 (22)	0	52 (20)	7 (3)

Listed are all adverse events that occurred during the treatment period and up to 90 days after the last dose of study treatment or up to the start of any subsequent therapy (whichever occurred first)

*EP* platinum-etoposide

^a^Includes patients who permanently discontinued at least one study treatment

^b^An immune-mediated adverse event was defined as an event that was associated with drug exposure and was consistent with an immune-mediated mechanism of action, where there was no clear alternate etiology and the event required treatment with systemic corticosteroids or other immunosuppressants and/or, for specific endocrine events, endocrine therapy

**Table 5:** Adverse events of any cause with an incidence of ≥ 15% in either arm and all grade 3 or 4 events

	Durvalumab + EP (*n* = 18)		EP (*n* = 16)	
	Any grade	Grade 3 or 4	Any grade	Grade 3 or 4
Any event, *n* (%)	18 (100)	14 (78)	16 (100)	15 (94)
Constipation	13 (72)	0	7 (44)	0
Nausea	8 (44)	0	10 (63)	1 (6)
Neutropenia	6 (33)	6 (33)	7 (44)	7 (44)
Alopecia	6 (33)	0	6 (38)	0
Anemia	4 (22)	1 (6)	6 (38)	2 (13)
Hiccups	5 (28)	0	5 (31)	0
Neutrophil count decreased	5 (28)	4 (22)	5 (31)	3 (19)
Decreased appetite	6 (33)	2 (11)	3 (19)	1 (6)
Febrile neutropenia	6 (33)	6 (33)	3 (19)	3 (19)
Insomnia	4 (22)	0	4 (25)	0
White blood cell count decreased	2 (11)	1 (6)	6 (38)	4 (25)
Dry skin	3 (17)	0	3 (19)	0
Malaise	4 (22)	0	2 (13)	0
Pyrexia	4 (22)	0	2 (13)	0
Hyponatremia	2 (11)	0	3 (19)	0
Stomatitis	2 (11)	0	3 (19)	0
Vomiting	2 (11)	0	3 (19)	0
Headache	2 (11)	1 (6)	2 (13)	0
Peripheral sensory neuropathy	1 (6)	0	3 (19)	0
Platelet count decreased	2 (11)	0	2 (13)	1 (6)
Diabetes mellitus	1 (6)	1 (6)	2 (13)	0
Vasculitis	0	0	3 (19)	0
Alanine aminotransferase increased	0	0	2 (13)	1 (6)
Bacterial infection	2 (11)	1 (6)	0	0
Hyperglycemia	2 (11)	1 (6)	0	0
Pneumonia	1 (6)	1 (6)	1 (6)	1 (6)
Acute myocardial infarction	0	0	1 (6)	1 (6)
Aspartate aminotransferase increased	0	0	1 (6)	1 (6)
Cardiac tamponade	1 (6)	1 (6)	0	0
Embolism arterial	1 (6)	1 (6)	0	0
Hypertension	1 (6)	1 (6)	0	0
Hypocalcemia	0	0	1 (6)	1 (6)
Loss of consciousness	0	0	1 (6)	1 (6)
Lung infection	0	0	1 (6)	1 (6)
Sepsis	0	0	1 (6)	1 (6)
Syncope	0	0	1 (6)	1 (6)
Type 1 diabetes mellitus	1 (6)	1 (6)	0	0

Listed are all adverse events that occurred during the treatment period and up to 90 days after the last dose of study treatment or up to the start of any subsequent therapy (whichever occurred first). The events are listed in descending order of frequency for any-grade events across both the treatment arms

*EP* platinum-etoposide

There were no AEs leading to discontinuation of treatment, or to death, in either study arm. Immune-mediated AEs (imAEs) of any grade were reported in 4 (22%) patients in the durvalumab plus EP arm; no imAEs were reported in the EP arm (Table 4). One imAE of type 1 diabetes mellitus was grade 3. The remaining imAEs were grade 1 or 2 in severity and comprised one case each of hyperthyroidism, hypothyroidism, and interstitial lung disease (Table 6). imAEs were managed with corticosteroids or endocrine therapy per toxicity management guidelines.

**Table 6:** Immune-mediated adverse events in the durvalumab plus EP arm (*n* = 18)

	Any grade	Grade 3^a^	Intervention	Resolved
Any imAE, *n* (%)^b^	4 (22)	1 (6)	–	–
Hyperthyroidism	1 (6)	0	Endocrine therapy	Yes
Hypothyroidism	1 (6)	0	Endocrine therapy	No
Interstitial lung disease	1 (6)	0	Systemic corticosteroid/high-dose steroid	No
Type 1 diabetes mellitus	1 (6)	1 (6)	Endocrine therapy	No

No imAEs were reported among patients treated with EP alone

^a^No grade 4 imAEs were reported

^b^An immune-mediated adverse event was defined as an event that was associated with drug exposure and was consistent with an immune-mediated mechanism of action, where there was no clear alternate etiology and the event required treatment with systemic corticosteroids or other immunosuppressants and/or, for specific endocrine events, endocrine therapy

*EP* platinum-etoposide, *imAE* immune-mediated adverse event

## Discussion

In this preplanned subgroup analysis of the CASPIAN study, efficacy and safety in the Japan subgroup were broadly consistent with the global study findings [7], despite the small sample size. Numerically longer OS was observed with durvalumab plus EP compared with EP alone in patients with ES-SCLC recruited in Japan (HR 0.77 [95% CI 0.26‒2.26]). This survival benefit was consistent with the results in the global CASPIAN study population (OS HR of 0.73 [95% CI 0.59‒0.91; *p* = 0.0047]) [7]. In the Japan subgroup, 6-month PFS rates and objective response rates also numerically favored durvalumab plus EP compared with EP alone, and durvalumab plus EP was generally well tolerated with no AEs leading to death or discontinuation.

Numerically longer median OS was seen in both treatment arms in the Japan subgroup compared with the global population (durvalumab plus EP: not reached vs 13.0 months; EP: 15.2 months vs 10.3 months); 12-month OS rates were similarly higher in the Japan subgroup versus the global population (durvalumab plus EP: 72% vs 54%; EP: 60% vs 40%) [7]. Cisplatin use was higher in the Japan subgroup than in the global population (44% [15/34] vs 25% [132/537]). This is consistent with real-world data showing that 42% of Japanese patients with ES-SCLC received cisplatin-based chemotherapy as first-line treatment between 2014 and 2016 (compared with ~ 27% of patients in the USA) [4]. Although there are no data available to suggest a difference in efficacy between carboplatin and cisplatin in ES-SCLC, these data highlight the importance of the flexibility in the choice of platinum agent offered in the CASPIAN study. This is in contrast to the IMpower133 study, in which carboplatin-etoposide was the only chemotherapy regimen administered in combination with first-line atezolizumab in patients with ES-SCLC [6, 18].

In CASPIAN, a higher proportion of patients received subsequent systemic anticancer therapy in the Japan subgroup compared with the global population (overall rate across both arms 76% [26/34] vs 43% [232/537]), which may have contributed to the longer median OS in the Japan subgroup in both arms when compared with the global population. Patients in the Japan subgroup were also more likely than the global population to have had at least two subsequent lines of therapy (38% [13/34] vs 13% [72/537]). A similar observation was made in the IMpower133 study: in the Japanese subgroup, the median OS was longer in both treatment arms and the proportion of patients receiving subsequent therapy was greater compared with the global population [6, 18].

The addition of durvalumab to EP was generally well tolerated and there were no AEs leading to death or discontinuation in the Japan subgroup of CASPIAN. The overall safety profile was consistent with the global population and the known safety profiles for each individual agent. The incidence of imAEs in the Japan subgroup was similar to that in the global population. The rate of pneumonitis imAEs was low in the global population (3% in the durvalumab plus EP arm) [7], and in the Japan subgroup only one patient (6% in the durvalumab plus EP arm) had a pneumonitis or interstitial lung disease event, which was low grade in severity. Due to the small sample size in the Japan subgroup (a 3% incidence equates to < 1 patient in the durvalumab plus EP arm), it is not possible to compare the incidence of pneumonitis with the global population in a meaningful way.

While the incidences of grade 3 or 4 AEs and serious AEs were numerically higher across both treatment arms in the Japan subgroup compared with the global population [7], which may reflect local medical practice, the lack of AEs leading to treatment discontinuation or death in the Japan subgroup suggests that severe and serious AEs were generally manageable. The higher rate of grade 3 or 4 AEs in the Japan subgroup was driven by higher rates of neutropenia and febrile neutropenia. The rates of febrile neutropenia reported here are generally consistent with those reported previously for patients treated with EP in Japan [22]. An increased incidence of grade 3 or 4 events was also reported in the Japanese subgroup in IMpower133 compared with non-Japanese patients [18] and the entire population [6]; the authors attributed this to increased hematotoxicity in Japanese patients receiving chemotherapy. This is also consistent with previously reported differences in the rate of severe hematological toxicity associated with chemotherapy between Japanese and non-Japanese patients with lung cancer [10, 11, 22].

Limitations of this subgroup analysis include, primarily, the small sample size and the fact that the analysis was not powered for efficacy comparisons. The maturity in the Japan subgroup was less than in the global population (41% vs 63%) and the median follow-up shorter (12.5 vs 14.2 months), which is likely a consequence of the small sample size and the later date of enrollment of the first patient in Japan. The data cut-off date for the interim analysis was determined by the number of events in the global population, per protocol, and while the follow-up was appropriate for the global analysis, it was not ideal in this subgroup, with the median OS not reached in the durvalumab plus EP arm. In addition, interpretation of the efficacy and safety data within the Japan subgroup may be limited by the potential impact of imbalances in patient characteristics and use of cisplatin between the treatment arms, as the analysis was exploratory and randomization in the Japan subgroup was not stratified. Despite these limitations, our analysis provides valuable insights into the efficacy and safety of durvalumab in the Japanese population, building on previous reports of durvalumab in Japanese patients with advanced solid tumors [17] or with stage 3, unresectable NSCLC [23, 24].

In conclusion, the addition of durvalumab to EP was effective and well tolerated in Japanese patients with ES-SCLC. Despite the small size of the Japan subgroup in CASPIAN, results were generally consistent with the global population.

## Supplementary Information

Below is the link to the electronic supplementary material.

Supplementary file1 (PDF 45 kb)
