# Supplementary material for: First-line durvalumab plus platinum-etoposide in extensive-stage small-cell lung cancer: CASPIAN Japan subgroup analysis
Source: Int J Clin Oncol. 2021 Apr 7;26(6):1073–82. doi: 10.1007/s10147-021-01899-8 (PMC8134304; doi:10.1007/s10147-021-01899-8)
Supplement: Supplementary file 1 — Supplementary file1 (PDF 45 kb) [file 10147_2021_1899_MOESM1_ESM.pdf]

Supplementary material

Supplementary Table 1 Subsequent anticancer therapy

	Durvalumab + EP (*n* = 18)	EP (*n* = 16)
Patients receiving any subsequent systemic therapy, *n* (%)	13 (72)	13 (81)
Chemotherapy, *n* (%)[a]	13 (72)	11 (69)
Single agent	12 (67)	9 (56)
Platinum doublet	5 (28)	4 (25)
Immunotherapy, *n* (%)[a]	0	1 (6)
Immunotherapy + chemotherapy		1 (6)
Other systemic therapy, *n* (%)[a]	0	2 (13)[b]
Line of treatment, *n* (%)[a]		
Patients receiving ≥1 subsequent line	13 (72)	13 (81)
Patients receiving ≥2 subsequent lines	8 (44)	5 (31)
Patients receiving >2 subsequent lines	4 (22)	2 (13)

[a]Patients with regimens or lines of therapy in more than one category are counted once in each of those categories
[b]Both patients received an investigational product
EP platinum-etoposide

Supplementary Table 2 Serious adverse events of any cause

	Durvalumab + EP<br>(*n* = 18)	EP<br>(*n* = 16)
Any serious event, *n* (%)	8 (44)	8 (50)
Febrile neutropenia	2 (11)	2 (13)
Acute myocardial infarction	0	1 (6)
Cardiac dysfunction	1 (6)	0
Diabetes mellitus	1 (6)	0
Embolism arterial	1 (6)	0
Herpes zoster	1 (6)	0
Ileus	0	1 (6)
Interstitial lung disease	1 (6)	0
Loss of consciousness	0	1 (6)
Lung infection	0	1 (6)
Neutropenia	0	1 (6)
Pneumonia	0	1 (6)
Rectal cancer	1 (6)	0
Sepsis	0	1 (6)
Syncope	0	1 (6)
Transient ischemic attack	1 (6)	0
Type 1 diabetes mellitus	1 (6)	0

Listed are all serious adverse events that occurred during the treatment period and up to 90 days after the last dose of study treatment or up to the start of any subsequent therapy (whichever occurred first). A serious adverse event was defined as an event that met one of the following criteria: resulted in death; was immediately life threatening; required hospitalization or prolongation of existing hospitalization; resulted in persistent or significant disability or incapacity; was a congenital abnormality or birth defect; or was considered an important medical event that could jeopardize the patient or require medical intervention in order to prevent one of the previous outcomes
EP platinum-etoposide
